# Clinical efficacy of electroacupuncture on pelvic floor function in women with stress urinary incontinence: a randomized sham-controlled trial protocol

**DOI:** 10.3389/fgwh.2025.1713321

**Published:** 2026-01-29

**Authors:** Shuren Ming, Jiaxin Yang, Bingli Chen, Juanjuan Li, Wenguang Hou, Yuelai Chen

**Affiliations:** 1 Longhua Hospital Shanghai University of Traditional Chinese Medicine, Shanghai, China; 2 Yueyang Hospital of Integrated Traditional Chinese and Western Medicine, Shanghai University of Traditional Chinese Medicine, Shanghai, China

**Keywords:** acupuncture, non-pharmacological treatment, pelvic floor disorders, randomized controlled trial, study protocol, surface electromyography, transperineal ultrasound, women's health

## Abstract

**Background:**

Stress urinary incontinence (SUI) is a prevalent condition among adult women, significantly impairing quality of life. Patients with SUI often exhibit abnormal pelvic floor muscle function, which can be comprehensively assessed using surface electromyography (sEMG) and transperineal ultrasound (TPUS). Electroacupuncture (EA) is an effective treatment for SUI, and multiple studies have confirmed its clinical efficacy; however, objective evidence regarding its impact on pelvic floor muscle function remains limited. This study aims to evaluate the clinical efficacy of EA applied to lower abdominal acupoints for SUI and to explore its impact on pelvic floor function, thereby providing mechanistic insights into EA's therapeutic effect on mild to moderate SUI.

**Methods:**

This is a randomized, single-blind trial with a 1:1 allocation ratio. A total of 64 participants will be enrolled. Following screening, eligible female patients with SUI will be randomly allocated to either the EA group (*n* = 32) or the sham EA group (*n* = 32), receiving EA or sham EA treatment, respectively. The intervention period will last 6 weeks. The primary outcome is the change in urine leakage, measured by the 1-hour pad test, from baseline to week 6. Secondary outcomes include: the incontinence episode frequency (IEF); the International Consultation on Incontinence Questionnaire-Short Form (ICIQ-SF) score; SUI severity; weekly urine pad usage; participants' self-assessment of therapeutic effect; pelvic floor muscle sEMG parameters; TPUS measurements; discomfort during treatment assessed by a Visual Analog Scale (VAS); participants' acceptability; and intervention-related adverse events.

**Conclusion:**

By comprehensively evaluating symptom improvement, temporal effects, and pelvic floor functional changes following EA treatment for SUI, this study aims to systematically elucidate the therapeutic effects of EA and its mechanism of action on the pelvic floor. The findings are expected to provide robust evidence to support the clinical application of EA for SUI.

**Clinical Trial Registration:**

https://itmctr.ccebtcm.org.cn/mgt/project/view/-6004709097458762939, identifier ITMCTR2024000151.

## 1 Introduction

Stress urinary incontinence (SUI) is a highly prevalent condition among adult women. The International Continence Society (ICS) defines SUI as the complaint of involuntary urine leakage upon effort or exertion, such as laughing, sneezing, or coughing (1). Epidemiological data indicate a high incidence of SUI. The median global prevalence of urinary Incontinence in women is 27.6% (range: 4.8%–58.4%), with SUI accounting for over 50% of cases (2). SUI severely impacts patients’ social activities, physical exercise, and sexual life, leading to psychological issues such as anxiety and depression, as well as social isolation. Consequently, it significantly reduces quality of life (3) and imposes a substantial economic burden on society. SUI is closely associated with impairments in pelvic floor structure and function. Patients often exhibit a weakened urethral mucosal seal, inadequate urethral sphincter and pelvic floor muscle strength, laxity of fascia and ligaments, and other functional or structural abnormalities of the pelvic floor. Insufficient pelvic floor support can lead to hypermobility of the bladder neck and proximal urethra. When abdominal pressure increases, bladder pressure may exceed urethral closure pressure, resulting in urine leakage (4).

Current treatment options for SUI primarily include behavioral interventions, pelvic floor muscle training (PFMT), biofeedback, pharmacotherapy, and surgery (5). PFMT is the first-line treatment for mild to moderate SUI; however, it requires patients to adhere to 3–6 months of training to achieve potential efficacy. It also demands professional guidance and supervision, which can challenge patient compliance (6). Electroacupuncture has gained recognition as a promising non-pharmacological treatment for SUI. Studies have demonstrated its efficacy in reducing urine leakage and improving quality of life (7, 8). Acupuncture at lower abdominal acupoints is widely employed in SUI treatment (9). Notably, it has been shown to immediately reduce the mobility of the bladder neck and proximal urethra, thereby improving pelvic floor function (10).

The therapeutic mechanisms of electroacupuncture for SUI are considered to involve multiple pathways. Firstly, it may modulate spinal and central reflex arcs to enhance urethral sphincter control and improve pelvic floor muscle function (7, 11). Secondly, it is thought to promote local blood circulation and nutrient supply, thereby aiding in the recovery of muscle elasticity and strength (12, 13). Additionally, electroacupuncture may facilitate tissue repair by modulating collagen metabolism in pelvic supportive structures (14, 15). These multifaceted actions highlight its potential as a comprehensive therapy for SUI. Despite its established clinical efficacy, significant gaps remain in the objective characterization of EA's impact on pelvic floor physiology. Many prior studies lack a systematic integration of objective functional and structural assessments. Furthermore, when EA is combined with pelvic floor muscle training, the specific effects attributable to EA alone are often unclear (7, 16).

Therefore, this sham-controlled trial aims to rigorously evaluate the efficacy of EA as a monotherapy for SUI and to investigate its specific effects on pelvic floor muscle function and structure, thereby providing novel mechanistic insights.

## 2 Methods and analysis

### 2.1 Study design

**Figure 1:**
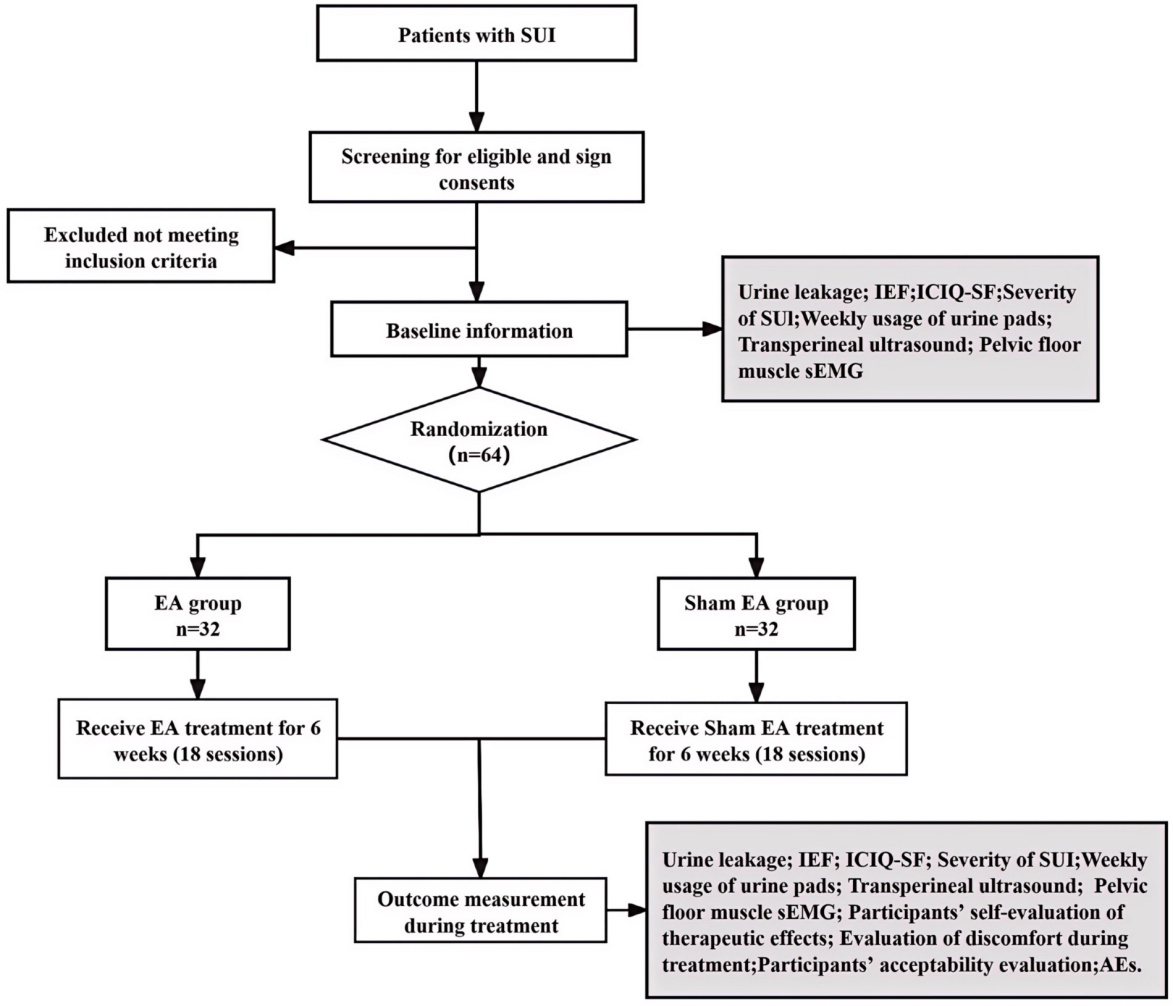
Flowchart. SUI, stress urinary Incontinence; IEF, incontinence episode frequency; sEMG, surface electromyography; ICIQ-SF, International Consultation on Incontinence Questionnaire-Short Form; AEs, adverse events.

This study is a randomized, single-blind, controlled trial. The study will be conducted in the acupuncture department of Yueyang Hospital of Integrated Traditional Chinese and Western Medicine, Shanghai University of Traditional Chinese Medicine, China. The study protocol has been approved by the Medical Ethics Committee of Yueyang Hospital of Integrated Traditional Chinese and Western Medicine, Shanghai University of Traditional Chinese Medicine (Approval No. 2024-039) and is registered with the International Traditional Medicine Clinical Trial Registry (ITMCTR2024000151). The protocol adheres to the Standard Protocol Items: Recommendations for Intervention Trials (SPIRIT) 2013 guidelines (17). The study flowchart is presented in Figure 1, and the enrollment, interventions, and assessments schedule is outlined in Table 1.

**Table 1:** Schedule of recruitment, interventions and assessments.

Study period	Enrolment	Intervention					
Time point	Week 0	Week 1	Week 2	Week 3	Week 4	Week 5	Week 6
Inclusion/exclusion criteria	X						
Sign informed consent	X						
Baseline information collection	X						
Randomization	X						
Intervention							
Electroacupuncture		X					
Sham electroacupuncture		X					
Primary outcome							
Urine leakage	X						X
Secondary outcomes							
Incontinence episode frequency	X		X		X		X
ICIQ-SF	X		X		X		X
Severity of SUI	X		X		X		X
Weekly usage of urine pads	X		X		X		X
Participants’ self-evaluation of therapeutic effects			X		X		X
Pelvic floor surface Electromyography	X						X
Transperineal ultrasound	X						X
Safety evaluation		X	X	X	X	X	X
Evaluation of discomfort during treatment		X		X			
Participants’ acceptability evaluation		X		X			

SUI, stress urinary incontinence; ICIQ-SF, International Consultation on Incontinence Questionnaire-Short Form.

### 2.2 Participant and consumer involvement

The inclusion criteria are as follows: diagnosis of mild or moderate SUI in women (18, 19); age between 40 and 75 years. All participants will provide written informed consent before enrollment. Exclusion criteria include: urge urinary Incontinence, mixed urinary Incontinence, or overflow urinary Incontinence; history of surgical treatment for urinary Incontinence or pelvic floor surgery; genital prolapse of stage II or greater; symptomatic urinary tract infection; postvoid residual urine volume > 30 mL; maximum urinary flow rate < 20 mL/s; inability or limited ability to walk, climb stairs, or run; continuous use of medications affecting bladder function or ongoing specialized treatment for SUI; severe cardiac, cerebral, hepatic, renal, hematopoietic system, or psychiatric diseases; diabetes mellitus, multiple system atrophy, cauda equina lesions, or spinal cord pathologies; pregnancy, lactation, or intention to become pregnant during the trial period; presence of a cardiac pacemaker, metal allergy, or severe needle phobia. The withdrawal criteria are as follows: experience of severe complications or other serious illnesses that preclude continued participation; participant's request to withdraw from the trial. Data will be analyzed according to the intention-to-treat (ITT) principle.

### 2.3 Operator qualifications and standardization

Two licensed acupuncturists will perform all acupuncture procedures, each with at least five years of clinical experience and a nationally recognized acupuncture practice certificate. Before the study commencement, all operators will undergo a one-week standardized training program covering acupoint localization, needle insertion depth, manipulation techniques, and safety protocols. Inter-operator consistency will be assessed through simulated treatment sessions, requiring at least 85% agreement on key parameters (e.g., acupoint localization accuracy, insertion depth, and manipulation frequency).

### 2.4 Interventions

Before treatment, participants will be instructed to empty their bladders and assume a supine position. All treatments will be administered following skin disinfection. Disposable sterile acupuncture needles (Guizhou Andi Pharmaceutical Equipment Co., Ltd.; sizes: 0.30 mm × 75 mm and 0.30 mm × 25 mm) will be used. Participants will receive three weekly treatment sessions (on alternate days), 30 min per treatment session, totaling 18 sessions over 6 weeks.

#### 2.4.1 EA group

**Figure 2:**
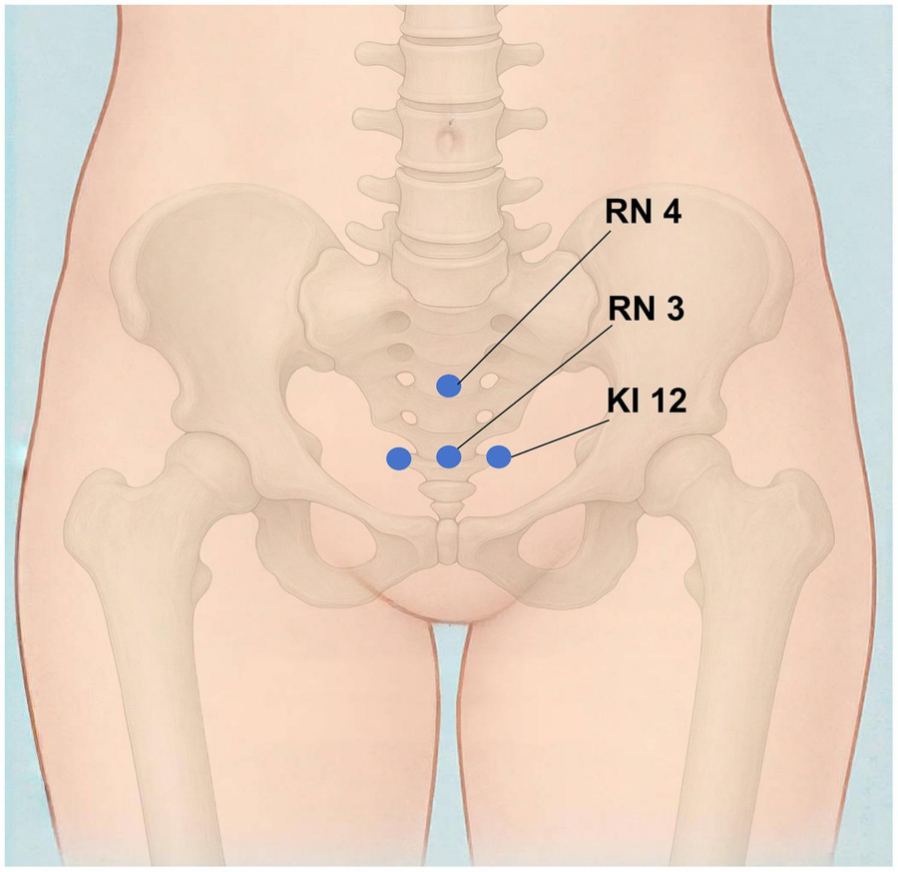
Locations of acupoints. RN3: Zhongji; RN4: Guanyuan; KI12: Dahe.

Sterile adhesive pads will be positioned at the acupoints Guanyuan (RN4), Zhongji (RN3), and Dahe (KI12) (Figure 2). A 0.30 mm × 75 mm needle will be inserted through the pad at an angle of 30° to 45° toward the perineum, reaching a depth of 40–50 mm. The needle will then be rotated gently and evenly at approximately 60 revolutions per minute with an amplitude of 180°–360° to elicit the *deqi* sensation, characterized by soreness, numbness, or distension radiating toward the bladder, perineum, or urethra. Subsequently, electroacupuncture stimulation will be applied using a continuous wave at 50 Hz. The current intensity will be set between 1 mA and 5 mA and gradually increased until a slight, painless tremor is observed in the tissues surrounding the acupoint. One pair of electrodes will connect the left KI12 and RN3 acupoints, and another pair will connect the right KI12 and RN4 acupoints.

#### 2.4.2 Sham EA group

Sterile adhesive pads will be placed at the same acupoint locations (RN3, RN4, KI12). A blunted tip needle (0.30 mm × 25 mm) will be placed onto the pad at a 30 °–45 ° angle without penetrating the skin. The *deqi* sensation will not be elicited (Table 2). The electroacupuncture apparatus will be identical in appearance to that used in the EA group, but will have its internal circuits disconnected, ensuring no current is delivered. The device display will indicate 50 Hz and 1–5 mA settings, but participants will not perceive any electrical stimulation. Electrodes will be connected superficially in a comparable configuration: between the left KI12 and RN3, and the right KI12 and RN4 acupoints.

**Table 2:** Locations and manipulations of acupoints.

Acupoint name	Location description	Manipulation details (EA group)	Manipulation details (sham EA group)
Zhongji (RN3)	On the anterior midline, 4 cun inferior to the umbilicus.	Following skin disinfection, a sterile filiform needle (0.30 mm × 75 mm) is inserted at each acupoint at a 30 °–45 ° angle toward the perineum to a 40–50 mm depth. Manual needle manipulation is performed to elicit the *deqi* sensation. Subsequently, electrodes are connected (left KI12-RN3 and right KI12-RN4) and electrical stimulation is applied (continuous wave, 50 Hz, 1–5 mA, adjustable to a painless muscle twitch).	Following skin disinfection, a sterile blunted tip needle (0.25 mm × 25 mm) is placed at the same angle onto the pad, but does not penetrate the skin. No manipulation is performed, and *deqi* is not elicited. The sham device, identical in appearance, is connected similarly but delivers no current.
Guanyuan (RN4)	On the anterior midline, 3 cun inferior to the umbilicus.		
Dahe (KI12)	0.5 cun lateral to the anterior midline, 4 cun inferior to the umbilicus.		

Both interventions will be administered in accordance with the methods described by Liu et al. (7)and the SHARE guidelines (20).

### 2.5 Outcome measures

#### 2.5.1 Primary outcome

The change in urine leakage, as measured by the 1-hour pad test, from baseline to week 6. The test procedure will follow: the participant will wear a pre-weighed pad, drink 500 mL of water within 15 min, and perform a series of activities. These activities include walking for 30 min (which incorporates stair climbing), repeatedly sitting down and standing up, vigorous coughing, running in place, bending to pick up a small object from the floor, and washing hands under running water. The pad will then be reweighed to quantify the urine leakage (21). The assessment will be postponed or rescheduled if the participant is menstruating or experiences an episode of severe coughing.

#### 2.5.2 Secondary outcomes

1. Incontinence episode frequency (IEF): Mean IEF over 72 h will be recorded in a bladder diary. Assessments will be performed at baseline, week 2, week 4, and week 6. Change from baseline will be calculated.
2. ICIQ-SF score: The International Consultation on Incontinence Questionnaire-Short Form (ICIQ-SF) will be used to retrospectively evaluate the frequency, volume of Incontinence, and impact on quality of life over the preceding four weeks. The validated Chinese version will be administered (22). The questionnaire includes three scored items (assessing the frequency of urinary Incontinence, the amount of leakage, and the degree of interference with quality of life) and one non-scored self-diagnosis item (used to provide additional qualitative information). The total score ranges from 0 to 21, with higher scores indicating greater symptom severity (23). Scores will be collected at baseline, week 2, week 4, and week 6.
3. Severity of SUI: The overall severity of urinary leakage was evaluated based on patient-recorded 72-hour bladder diaries, which documented episodes occurring under normal circumstances (i.e., excluding vigorous coughing, exercise, or heavy lifting). Severity was categorized as follows: Mild: leakage of a few drops; Moderate: leakage that partially soaks a pad; Severe: leakage that thoroughly soaks a pad in a single episode. The most severe episode encountered is recorded (7). To enhance objectivity, all participants will be provided with standardized urinary pads (Brand: TENA, Size: 375 mm × 135 mm, Absorption: 100 mL). Additionally, we will record the 72-hour cumulative pad weight (difference between wet and dry weight using a precision scale  ± 0.1 g) as a quantitative measure of leakage severity. Participants can store pads in labelled zipper bags for weighing, reporting daily and 72-hour total leakage amounts. The severity of stress urinary incontinence will be assessed by trained outcome assessors who are blinded to the participants' group allocation. Assessments will be conducted at baseline, week 2, week 4, and week 6.
4. Weekly usage of urine pads: Participants will maintain a daily diary to record the number of pads used. The change from baseline in the average number of pads used per 2 weeks will be calculated. To provide objective supplementation, we will also derive two metrics from standardized pad weights: weekly total pad weight (g) and mean leakage per pad (g/pad). Provide participants with identical pads and labeled bags. Each pad's dry weight should be pre-measured and labeled at the center (ID and tare), with participants returning only the wet pads. All weighings should be performed on the same scale, by the same trained personnel, within a fixed time window, with daily calibration logs maintained. Data will be collected at baseline, week 2, week 4, and week 6.
5. Participants' self-evaluation of therapeutic effects: This subjective satisfaction rating assesses participants’ perception of treatment benefit. Participants will be asked, “Has the treatment helped you control your urine leakage?” Responses will be rated on a 4-point scale: 0 = not helpful at all; 1 = slightly helpful; 2 = moderately helpful; 3 = very helpful (7). Evaluations will be performed at week 2, week 4, and week 6.
6. Pelvic floor muscle surface EMG (sEMG): Parameters will be assessed at baseline and week 6 using the standard Glazer protocol with participants in the lithotomy position (24). The evaluated parameters include: pre-resting baseline tone; amplitude of phasic (Type II fast-twitch) muscle contractions (calculated as the mean amplitude of five rapid contractions); amplitude of tonic (Type I slow-twitch) muscle contractions (calculated as the mean amplitude of five 10-second sustained contractions); endurance of Type I muscles (mean amplitude during a single 60-second sustained contraction); and post-resting baseline tone. Changes in the EMG amplitude for both Type I and Type II muscle fibers from baseline to week 6 will be analyzed.
7. Transperineal ultrasound (TPUS): TPUS assessments will be performed at baseline and week 6 with participants in the lithotomy position. Parameters to be measured include: bladder neck mobility, urethral rotation angle, retrovesical angle (posterior bladder angle), position of the lowest point of the bladder (bladder neck descent), urethral funneling, and the type of cystocele (bladder prolapse) at rest and during the Valsalva maneuver (25).
8. Evaluation of discomfort during treatment: Discomfort experienced during the acupuncture session will be evaluated using a Visual Analog Scale (VAS) ranging from 0 cm (indicating no discomfort) to 10 cm (indicating severe discomfort). The average VAS score recorded within 5 min after the first and ninth treatments will be used for analysis. If a score from one session is missing, the available score from the other session will be used (7).
9. Participants' acceptability evaluation: The acceptability of the treatment procedure will be rated on a 5-point scale: 0 = very difficult to accept; 1 = somewhat difficult to accept; 2 = acceptable; 3 = easy to accept; 4 = very easy to accept. The average score will be calculated within 5 min after the first and ninth treatments. If a score from one session is unavailable, the score from the remaining session will be utilized (7).

#### 2.5.3 Safety evaluation

All adverse events (AEs) will be recorded in the Case Report Form (CRF). Details to be documented include the date of onset and resolution, severity, relationship to the intervention, and measures taken for management. Treatment-related AEs may include, but are not limited to: dizziness, fainting, abscess formation, subcutaneous hematoma, local infection, and other discomforts such as fatigue or palpitations. Any occurring AEs will be managed appropriately. Serious AEs will be reported immediately to the Data and Safety Monitoring Board (DSMB), which will assess their relatedness to the intervention and recommend continuing the study. The frequency and percentage of participants experiencing AEs in each group will be calculated.

### 2.6 Data analysis

#### 2.6.1 Sample size calculation

The sample size was calculated for a superiority trial comparing two independent means, with a 1:1 allocation ratio, a two-sided *α* of 0.05 (*Z_α/2_* = 1.96), and a power (1-*β*) of 80%(*Z_β_* = 0.84). Based on data from our previous research, the anticipated change in urine leakage (1-hour pad test) from baseline to week 6 was used for calculation: EA group mean *μ_t_* = 8.59 g, Sham EA group mean *μ_c_* = 4.81 g, standard deviation *σ* = 4.76 g, superiority margin *Δ* = 0. Using these parameters, the calculated sample size required per group was 25. Accounting for an estimated dropout rate of 20%, 64 participants (32 per group) will be required (26, 27).

$$n=\frac{2{\left(Z_{\alpha /2}+Z_{\beta }\right)}^{2}\;\times \sigma^{2}}{{\left(\mu_{t}\text{-}\mu_{c}\text{-}\Delta \right)}^{2}}$$

#### 2.6.2 Randomization

According to the enrollment order, eligible participants will be randomly assigned in a 1:1 ratio to either the EA or the sham EA group. An independent statistician will generate the randomization sequence using SPSS software (version 26.0; IBM Corp., Armonk, NY, USA), employing a simple randomization procedure. The allocation sequence will be concealed using sequentially numbered, opaque, sealed envelopes (SNOSE) until baseline assessments are completed and the participant is assigned to a treatment group (27, 28).

#### 2.6.3 Blinding

This study is designed as a randomized, single-blind trial. Participants, outcome assessors, and data analysts will remain blinded throughout the trial. Due to the nature of the interventions, the acupuncturists administering the treatment cannot be blinded. To maintain blinding integrity, all research staff (except for the acupuncturists) will receive standardized training to prevent unintentional unblinding. The same assigned acupuncturist will administer all treatment sessions for a given participant to ensure consistency. After baseline data collection, the assigned acupuncturist will open the envelope to reveal the group-specific instructions. Based on the group assignment, real electroacupuncture and sham electroacupuncture procedures will be performed in separate consultation rooms. A dedicated appointment scheduling system will ensure that participants from different groups are treated at various times and do not encounter each other. This measure is implemented to prevent communication between participants from different groups, thereby safeguarding the blinding of participants and the validity of the study outcomes. The success of participant blinding will be assessed upon completion of the entire treatment period (before outcome assessment). Participants will be asked: “Which treatment do you believe you received?” with the following response options: (a) real electroacupuncture, (b) sham electroacupuncture, (c) unsure, or (d) do not care. The integrity of blinding will be quantitatively evaluated using Bang's blinding index (29).

#### 2.6.4 Statistical methods

Statistical analyses will be performed on the intention-to-treat (ITT) population and the per-protocol (PP) population. For the ITT analysis, missing data will be handled using multiple imputation by chained equations (MICE). All statistical analyses will be conducted using SPSS software (version 26.0; IBM Corp.). Results will be presented with 95% confidence intervals, and a two-sided *P* value of less than 0.05 will be considered statistically significant. Continuous variables normally distributed will be summarized as mean ± standard deviation, while those with a non-normal distribution will be summarized as median and interquartile range (IQR). Comparisons of continuous variables between the two groups will be performed using the independent samples *t*-test (for normal data) or the Mann–Whitney *U*-test (for non-normal data). Categorical data will be described as frequencies and percentages, and comparisons between groups will be made using the appropriate Chi-squared test or Fisher's exact test. For longitudinal analysis of outcomes measured over time, generalized estimating equations (GEE) or linear mixed-effects models will be employed to account for correlations within subjects and to examine time-by-group interaction effects. In secondary analyses, we will explore potential effect modification by menopausal status by including an interaction term in our statistical models. For the exploratory secondary outcomes, interpretation will focus on effect sizes and confidence intervals rather than unadjusted *P* values to avoid inflation of type I error due to multiple comparisons. No formal multiplicity adjustment will be applied to secondary endpoints.

## 3 Discussion

Stress urinary incontinence (SUI) is a highly prevalent condition imposing a substantial societal burden and significantly impairing the quality of life of affected women. Globally, the prevalence of urinary Incontinence in women exceeds 25%, and SUI constitutes the most prevalent subtype, accounting for more than half of all cases. Among Chinese adult women, the overall prevalence of SUI is 18.9%, with reported averages of 27.5% in urban areas and 32.5% in rural areas (2, 30). The prevalence of SUI increases with age, peaking in the 50–59 age group (28.2%) (31). Notably, mild and moderate SUI comprise approximately 90% of all cases (32), highlighting the large population that could benefit from effective nonsurgical interventions. SUI exerts multiple detrimental effects on patients' lives. Frequent urine leakage and the long-term reliance on protective pads severely disrupt daily activities, sleep, and social interactions.

Furthermore, the condition frequently precipitates psychological distress, including anxiety and depression (33). In addition to the personal suffering, SUI imposes a heavy economic burden on patients and healthcare systems. For instance, in the United States, the average annual treatment cost for SUI can be as high as $50,000 (34). Despite this significant economic impact, treatment-seeking rates remain low, with only about 8.2% of affected individuals receiving care (35). Given the global trend of population ageing, the prevalence of SUI is projected to rise, consequently escalating the associated social and economic burdens.

Normal function of the pelvic floor muscles and their supporting structures is crucial for maintaining continence, and impairment of this function plays a central role in the pathogenesis of SUI. The pelvic floor muscles are composed primarily of two fiber types: Type I (slow-twitch) fibers, which constitute approximately 70% and provide tonic, sustained support; and Type II (fast-twitch) fibers, comprising about 30%, which are responsible for phasic, rapid contractions during sudden increases in intra-abdominal pressure (36). During a sudden rise in intra-abdominal pressure, the Type II fibers of the levator ani muscle and the external urethral sphincter contract rapidly. This reflex contraction elevates the pelvic diaphragm, reduces the urethral curvature, and increases urethral closure pressure, thereby preventing urine leakage (4, 37). In patients with SUI, weakened pelvic floor muscles and diminished supportive function compromise the ability to counteract sudden rises in intra-abdominal pressure, resulting in involuntary urine leakage (4). Studies have demonstrated that compared to healthy individuals, the pelvic floor muscles in SUI patients exhibit reduced activation, diminished maximal voluntary contraction strength, and impaired endurance during sustained contractions (38). Histopathological findings in SUI include decreased muscle fiber density, disorganized arrangement, significant atrophy, a reduced proportion of Type II fibers, and fibrous replacement in the levator ani muscle (39).

The amplitude of the signal obtained from surface electromyography (sEMG) of the pelvic floor muscles reflects the number of activated motor units. Thus, sEMG can be used to assess the contractile function of both Type I and Type II muscle fibers across the different phases of the Glazer assessment protocol in patients with SUI. The sEMG signal parameters correlate well with the intensity of pelvic floor muscle contractions and serve as an indirect measure of muscle strength. The average peak amplitude during both fast (phasic) and endurance (tonic) contractions is significantly lower in SUI patients than in healthy controls, indicating functional impairment of both Type I and Type II muscle fibers (40, 41).

Pelvic floor ultrasound, conversely, provides a direct assessment of structural changes in the pelvic floor, such as hypermobility of the bladder neck and proximal urethra. This imaging modality offers high practicality, providing reliable, reproducible, and accurate measurements. Key sonographic metrics include bladder neck descent (BND), urethral rotation angle (URA), retrovesical angle (RVA), the position of the lowest point of the bladder (BN-S), urethral funneling, cystocele, and the levator hiatal area (42). In SUI patients, the thickness of the puborectalis muscle is reduced both at rest and during maximal voluntary contraction. During a pelvic muscle contraction, the levator hiatal area often demonstrates inadequate closure, and bladder neck mobility increases, alongside urethrovesical angle alterations. These sonographic findings are consistent with diminished muscle elasticity and contractile function, compromising the ability to maintain adequate urethral closure pressure during stress (42–44).

Acupuncture has a long history of use in treating SUI. A standard treatment protocol has been established in contemporary clinical practice, primarily involving acupoints in the lower abdomen [e.g., Zhongji (RN3) and Guanyuan (RN4)] and the sacrococcygeal region [e.g., Huiyang (BL35) and Zhongliao (BL33)] (7, 8).In clinical practice, lower abdominal acupoints, particularly RN3 and RN4, are among the most frequently selected (9). Our team's previous pilot work demonstrated that electroacupuncture stimulation at RN3 can induce immediate improvements in pelvic floor structure among SUI patients, including reduced bladder neck descent (BND), changes in the urethral rotation and retrovesical angles at rest and during Valsalva, and elevation of the lowest point of the bladder (45). These findings suggest that electroacupuncture at lower abdominal acupoints may exert a positive regulatory effect on the pelvic floor support system, influencing its functional and structural properties.

Despite established efficacy, the mechanistic understanding of electroacupuncture in SUI remains incomplete. For instance, the landmark study by Liu et al. (7) provided high-level evidence for the efficacy of electroacupuncture in SUI, demonstrating its superiority over sham acupuncture in reducing urine leakage and improving quality of life. However, that study relied primarily on clinical efficacy indicators such as symptom questionnaires and pad tests, failing to reveal the accompanying changes in pelvic floor function and structure underlying the therapeutic effects. Furthermore, many current clinical studies tend to combine electroacupuncture with pelvic floor muscle training (PFMT) as a complex intervention for SUI (8, 16). While these studies confirm the effectiveness of the combined therapy, the comprehensive nature of the intervention makes it difficult to distinguish the specific contribution of electroacupuncture from that of PFMT, particularly obscuring the exact impact of electroacupuncture alone on pelvic floor muscle function. Our team's previous pilot studies (10, 45)preliminarily found that electroacupuncture could immediately improve ultrasound parameters like bladder neck mobility, suggesting its potential mechanism of action via regulating pelvic floor support structures. However, these studies focused on the immediate effect of single acupoint stimulation and lacked long-term observation, limiting the strength of the evidence. Additionally, most mechanistic studies of electroacupuncture for SUI remain at the animal experiment stage (11, 14, 15), and there is a lack of systematic evaluation integrating its physiological effects in humans, combining symptomatology, pelvic floor muscle electromyographic activity, and morphological changes.

To bridge this critical gap, namely, the lack of a systematic evaluation integrating subjective symptoms with objective physiological and structural measures, we designed this randomized, single-blind, sham-controlled trial in accordance with the SHARE guidelines (20) to rigorously assess the specific effects of EA. By employing a validated sham acupuncture device, this trial aims to isolate the specific therapeutic effects of electroacupuncture from non-specific effects, thereby enabling a comprehensive assessment of its efficacy, safety, and impact on pelvic floor function. However, a recognized limitation inherent to acupuncture trials, including this one, is the potential influence of non-specific effects, such as the placebo effect, patient expectations, and the therapeutic ritual. Although the sham acupuncture technique is designed to minimize placebo effects, the inability to blind the acupuncturists and the potential for patients to perceive differences in needle sensation between real and sham treatments remain essential potential sources of bias. To mitigate this risk, participants will receive interventions individually in separate rooms to prevent communication between them. Participants will be blinded to their group assignment to maintain objectivity.

Furthermore, concomitant pelvic floor muscle training is prohibited during the trial period to avoid confounding. If the trial demonstrates the superiority of electroacupuncture over sham treatment, the observed benefits may be attributed to mechanisms such as enhanced pelvic floor muscle tone, reduced muscle fatigue, and subsequent improvements in pelvic floor structure and continence function.

To quantitatively assess the success of blinding, we will use the Bang Blinding Index upon completion of the treatment period. Participants will be asked to guess their group assignment, and the results will be analyzed to determine the effectiveness of our blinding procedures. Successful blinding will help distinguish the specific effects of electroacupuncture from non-specific effects. Nevertheless, the inability to blind therapists and the potential for participants to discern treatment allocation remain inherent methodological limitations. These factors will be carefully considered when interpreting the trial results.

To minimize bias from participant dropout, we have preemptively developed comprehensive strategies, including offering flexible scheduling, allowing tolerant visit time windows, enhancing communication with participants, establishing an early warning system for dropouts, and strictly applying the intention-to-treat (ITT) principle in data analysis, combined with multiple imputation methods to handle missing data. Finally, for ethical considerations, participants can continue any pre-existing specialized SUI treatments; however, all such concomitant treatments will be meticulously recorded and considered as covariates in the statistical analysis to control for their potential confounding effects.

Several other limitations of this study should be acknowledged. First, the single-center design and recruitment of participants from a single institution may limit the sample representativeness and generalizability of the findings. Consequently, the external validity and generalizability of the conclusions may be limited and should be interpreted cautiously. Differences in healthcare settings and patient populations across regions may affect the widespread applicability of the findings. Although unified operator training was implemented to standardize the protocol, other single-center factors, such as the specific treatment environment and equipment, could introduce systematic bias. Second, although the sample size meets the basic requirements for the primary outcome, it is relatively modest compared to large multicenter trials (7). The relatively small sample size may reduce the statistical power for detecting significant effects in exploratory subgroup analyses and some secondary outcome measures. Third, as SUI is a chronic condition, understanding long-term outcomes is crucial. However, this study is limited to assessing short-term efficacy immediately post-intervention due to budgetary and logistical constraints. The lack of follow-up beyond 6 weeks precludes conclusions regarding the sustainability of treatment effects and the long-term efficacy of electroacupuncture. To address these limitations, future research should prioritize multicenter, large-sample randomized controlled trials that enroll more diverse populations and incorporate long-term follow-up (e.g., ≥6 months). Such studies are needed to comprehensively evaluate electroacupuncture's efficacy and sustainability for SUI and generate more robust evidence to guide its clinical application.
